# Measuring maternal near-miss in a middle-income country: assessing the use of WHO and sub-Saharan Africa maternal near-miss criteria in Namibia

**DOI:** 10.1080/16549716.2019.1646036

**Published:** 2019-08-13

**Authors:** Steffie Heemelaar, Leonard Kabongo, Taati Ithindi, Christian Luboya, Fidelis Munetsi, Ann-Kathrin Bauer, Amelie Dammann, Anna Drewes, Jelle Stekelenburg, Thomas van den Akker, Shonag Mackenzie

**Affiliations:** a Department of Obstetrics and Gynaecology, Katutura State Hospital, Windhoek, Namibia; b Department of Obstetrics, Leiden University Medical Center, Leiden, The Netherlands; c Department of Obstetrics and Gynaecology, Gobabis State Hospital, Gobabis, Namibia; d National Maternal Death, Stillbirth and Neonatal Death Review Committee, Ministry of Health and Social Services, Windhoek, Namibia; e Department of Obstetrics and Gynaecology, Rundu Intermediate Hospital, Rundu, Namibia; f Department of Obstetrics and Gynaecology, Okahandja State Hospital, Okahandja, Namibia; g Department of Health Science, Global Health, University of Groningen/University Medical Centre Groningen, Groningen, The Netherlands; h Department of Obstetrics and Gynaecology, Medical Center Leeuwarden, Leeuwarden, The Netherlands; i Department of Obstetrics and Gynaecology, University of Namibia, Windhoek, Namibia

**Keywords:** Maternal morbidity, severe maternal outcome, near-miss approach, morbidity registration, middle-income setting, sub-Saharan Africa maternal near-miss criteria, WHO maternal near-miss criteria

## Abstract

**Background**: Namibia, a middle-income country in sub-Saharan Africa (SSA), plans to use the Maternal Near Miss (MNM) approach. Adaptations of the World Health Organization (WHO) MNM defining criteria (‘WHO MNM criteria’) were previously proposed for low-income settings in sub-Saharan Africa (‘SSA MNM criteria’), but whether these adaptations are required in middle-income settings is unknown.

**Objective**: To establish MNM criteria suitable for use in Namibia, a middle-income country in SSA.

**Methods**: Cross-sectional study from 1 March 2018 to 31 May 2018 in four Namibian hospitals. Pregnant women or within 42 days of termination of pregnancy or birth, fulfilling at least one WHO or SSA MNM criterion were included. Records of women identified by either only WHO criteria or only SSA criteria were assessed in detail.

**Results**: 194 Women fulfilled any MNM criterion. WHO criteria identified 61 MNM, the SSA criteria 184 MNM. Of women who only fulfilled any of the unique SSA MNM criteria, 18 fulfilled the criterion ‘eclampsia’, one ‘uterine rupture’ and five ‘laparotomy’. These women were assessed to be MNM. Thresholds for blood transfusion to define MNM due to haemorrhage were two units in the SSA and five in WHO set. Two or three units were given to 95 women for mild/moderate haemorrhage or chronic anaemia who did not fulfil any WHO criterion and were not considered MNM. Fourteen women who were assessed to be MNM from severe haemorrhage received four units.

**Conclusions**: WHO MNM criteria may underestimate and SSA MNM criteria overestimate the prevalence of MNM in a middle-income country such as Namibia, where MNM criteria ‘in between’ may be more appropriate. Namibia opts to apply a modification of the WHO criteria, including eclampsia, uterine rupture, laparotomy and a lower threshold of four units of blood to define MNM. We recommend that other middle-income countries validate our criteria for their setting.

## Background

Maternal mortality has been reduced but remains high in sub-Saharan Africa (SSA) [1]. Even though sub-Saharan African countries aim for zero avoidable maternal deaths, many struggle to achieve substantial progress [1–5]. To further improve quality of maternity care, the World Health Organization (WHO) has promoted the Maternal Near-Miss (MNM) approach since 2004 [6]. An MNM is ‘a woman who nearly died but survived a pregnancy-related complication during pregnancy, birth or within 42 days after termination or delivery’ [6]. Women with an MNM event often share the same characteristics and risk factors as women who died, such as underlying medical or pregnancy-related conditions and delays in reaching and obtaining adequate health care. Much can be learned with regard to the functioning of the health system, and failing of the system, by analysing MNM and maternal deaths. However, MNM occurs in larger numbers and may be less threatening for health-care workers to discuss since these can be regarded as great saves [7,8].

Namibia has a high maternal mortality ratio of an estimated 385/100,000 livebirths in 2013 [9]. Even though it has a very large surface area, it is one of the least densely populated countries in the world with 2.8 people per square kilometer [10]. Annually, there are around 75,000 births [10]. To analyse and improve maternity care, Namibia is planning to apply the MNM approach. Adoption of a recognized set of MNM criteria will allow comparison across settings and is therefore preferred. Reports from other SSA countries indicated that the WHO MNM criteria (Appendix 1) may not be suitable for use in district hospitals in low-income settings, due to limited availability of laboratory tests, blood products and management options leading to under-registration of MNM [11,12]. Recently, a Delphi study was published proposing MNM criteria deemed suitable for hospitals in low-income settings in SSA [13]. The SSA criteria added several clinical criteria such as eclampsia, sepsis and uterine rupture and lowered the threshold for blood transfusion from five to two units of red cells (Appendix 1). For Namibia, classified by the World Bank as a sub-Saharan upper middle-income country, it is unclear which set of MNM criteria would be appropriate to use [14]. The national referral hospital in Windhoek, the capital of Namibia, has all the facilities to apply the WHO MNM criteria, but in district hospitals, several laboratory tests to identify organ failure or management options are not available. Nevertheless, Namibian district hospitals are generally better supplied compared to most district hospitals in low-income settings in SSA. The aim of this study was to establish MNM criteria suitable for use in all Namibian hospitals by applying both the WHO and the adapted SSA MNM criteria to a cohort of women in four Namibian hospitals.

## Methods

### Study setting and design

Participating facilities were the national referral hospital, Windhoek Hospital Complex (Hospital A), a large regional hospital, Rundu Intermediate Hospital (Hospital B), and two smaller district hospitals, Gobabis and Okahandja State Hospital (Hospital C and D). The hospitals were selected based on their characteristics, to obtain a representative sample of Namibian hospitals. Namibia has only three regional hospitals of which one is part of the hospital complex in the capital (Hospital A). Hospital B and the third regional hospital (not included) are similar in terms of number of births, available resources and catchment area. Of the 31 district hospitals, approximately half have more than 2000 births annually. Staff and resources are allocated accordingly. We selected two district hospitals representing a higher and lower-burden facility. Since Namibia is a large but sparsely populated country with long distances between health-care facilities, Hospital C was chosen since this facility is located in one of the least densely populated districts.

Hospital A is located in the capital, has almost 14,000 births annually, and is the only tertiary facility in Namibia for the entire population of 2.3 million [10]. There are three consultant obstetrician and gynaecologists. The Intensive Care Unit (ICU) has advanced equipment including ventilators and a dialysis machine. Sophisticated laboratory tests including pH and lactate measurements are available to identify and manage organ failure. Hospital B is located near the Angolan border, has just over 6,000 births annually and serves a population of approximately 350,000 [10]. There is one consultant gynaecologist, an ICU with mechanical ventilation and a well-supplied blood bank. The district hospitals have around 2500 and 1200 births, respectively, and there are no ICU facilities or consultants. Hospital C has basic haematology and chemistry laboratory tests available on site. Hospital D has no laboratory tests on site. Both district hospitals have a reliable supply of red cells for blood transfusion but no other blood products.

For this prospective cross-sectional study we included all pregnant women or within 42 days after termination of pregnancy or birth, regardless of gestational age, fulfilling at least one WHO or SSA MNM criterion. Furthermore, they had to present between 1 March 2018 and 31st of May 2018 to the participating facilities [6,13]. Maternal deaths, defined as death of a woman while pregnant or within 42 days of termination of pregnancy or birth, from any cause related to or aggravated by the pregnancy or its management, but not from accidental or incidental causes, were excluded [6].

### MNM identification criteria and sample size

The WHO and SSA criteria are presented in Appendix 1. The SSA criterion ‘Laparotomy other than caesarean section’ was modified to ‘Laparotomy other than caesarean section (CS) or ectopic pregnancy’, since we anticipated an over-reporting of ectopic pregnancies that were not necessarily an MNM. The threshold of transfusion of two units of blood was adopted from the SSA MNM criteria. The WHO MNM criterion defining MNM from haemorrhage has a threshold of five units of blood, so inclusion from two units of blood and above would allow further analysis of the women who received two, three or four units of blood to identify which threshold would most accurately define severe haemorrhage without over-reporting mild or moderate haemorrhage. In the proposed SSA criterion ‘severe pre-eclampsia with ICU admission’, ICU was not clearly defined. For our study, we decided to regard the ICU as a ward where mechanical ventilation and administration of continuous vasoactive drugs were possible, together with the continuous presence of a medical doctor.

At the time of design of the study, the performance of the SSA criteria was not yet assessed in clinical setting, rendering sample size calculations difficult. A large global study performed by the WHO found a prevalence of approximately 15 MNM per 1000 live births in countries with a maternal mortality ratio similar to that of Namibia [6,15]. Considering the lower threshold for transfusion and the addition of clinical criteria, we expected the SSA criteria to identify approximately twice as many MNM. To identify at least five MNM in the district hospitals, we set our study period at 3 months, with the possibility to extend it with another 3 months in case of insufficient inclusions in hospital C and D.

### Data collection and analysis

Doctors and nurses working in participating facilities, involved in the care of pregnant and/or postpartum women were trained in MNM identification and data collection prior to the study by members of the research team. A manual with information on data collection was present in each participating facility. MNM identification and data collection were supported in hospital A through daily visits to the maternity wards, gynaecology wards, acute care and ICU by AB, AD, AD, SH. On the respective wards, the admission books were screened for possible missed MNM. The register of the blood bank was screened for missed MNM by screening blood units delivered to the above-mentioned wards. Daily rounds and screening of blood bank registers were not feasible in the other hospitals due to budget and logistical restrictions. In hospitals B, C and D a doctor and two nurses were in charge of the project. These staff members were supervising MNM identification and data collection and were in close contact with the research team for support.

For the identified MNM, data were collected anonymously from medical records using a structured data collection tool, including socio-demographic characteristics, contributing factors, maternal and neonatal outcome, the main cause of MNM and long-term complications. All collected data were verified with medical records by either SH, LK, CL, FM, SM, who are medical doctors with at least 3 years of experience providing obstetric care in Namibia. We collected the total number of live births and maternal deaths that occurred within the facility during the study period.

Data were double entered and crosschecked in Epidata version 4.2. Data analysis consisted of frequencies of demographic and clinical variables. Data analysis was performed with SPSS version 22. All results are reported as numbers (n) and frequencies. Confidence intervals (CI) were calculated using the Clopper-Pearson exact test.

Severe maternal outcome (SMO) was defined as the number of MNM and maternal deaths combined. The MNM ratio was defined as the number of maternal near-miss per 1000 live births and the SMO ratio by the number of women with SMO per 1000 live births [6]. The confidence interval was calculated using Poisson distribution.

In order to establish MNM criteria which will identify all MNM in Namibian hospitals without over-reporting minor morbidities we did the following: for the women identified as an MNM by one of the unique criteria, medical records were reviewed by SH, LK, CL, FM, SM. Based on their clinical judgement it was decided for each unique criterion if it should be used for MNM identification in Namibia.

This study was reviewed and approved by the research unit of the Namibian Ministry of Health and Social Services. We followed the STROBE reporting guidelines.

## Results

In the three-month study period, 194 women were identified to be MNM according to the WHO and/or SSA criteria. Of these 194 MNM, 31 were identified through the blood bank register and ward registers of hospital A. Nine women were included by staff, but upon verification with medical records, it appeared these did not fulfil any MNM criterion and were excluded. Four of these nine women were included for ‘severe pre-eclampsia with ICU admission’, however these women were not admitted to an ICU department. Two women received one unit of blood for transfusion, whereas the threshold of the MNM criterion was at least two units of blood. Two women were included for sepsis but did not reach any of the clinical thresholds in the definition of the SSA criterion, Appendix 1. One woman was included for uterine rupture. However, only a pending uterine rupture was found with the lower uterine segment thin but intact.

Characteristics of the identified MNM are presented in Table 1. There were 32 (16.5%) teenage pregnancies and 60 (30.9%) women were primiparous. Chronic anaemia was present in 32 (16.5%) women. Obstructed labour was present in 18 (9.3%) women and 71 (36.6%) women gave birth by caesarean section. Miscarriage was present in 31 women, of whom 28 needed surgical evacuation, one women received medical treatment and two women had a spontaneous complete abortion.

**Table 1.** Characteristics of all maternal near-miss women.

Characteristics	(n = 194)	%
**Age**		
<20	32	16.5%
21–34	130	67.0%
≥35	32	16.5%
**Parity**		
Para 0	60	30.9%
Para 1–3	114	58.8%
≥4	18	9.3%
Unknown	2	1.0%
**Gestational age^a^**		
≤12 weeks	32	16.5%
13–26 weeks	25	12.9%
27–36 weeks	46	23.7%
≥37 weeks	80	41.2%
Unknown	11	5.7%
**Previous CS**		
No	155	79.9%
Yes	32	16.5%
Unknown	7	3.6%
**HIV status**		
Positive	18	9.3%
Negative	144	74.2%
Unknown	32	16.5%
**Chronic anemia**		
No	118	60.8%
Yes	32	16.5%
Unknown	44	22.7%
**Other comorbidities**		
Astma	1	0.5%
GDM	2	1.0%
Chronic hypertension	3	1.5%
TB	1	0.5%
Nephrotic syndrome	1	0.5%
Unknown	6	3.1%
**Pregnancy outcome**		
NVD	57	29.4%
Vacuum	3	1.5%
CS	71	36.6%
Laparotomy uterine rupture	2	1.0%
Miscarriage	31	16.0%
Ectopic	15	7.7%
Undelivered	14	7.2%
TOP	1	0.5%

NVD, normal vaginal delivery; CS, Caesarean section; TOP, termination of pregnancy; GDM, gestational diabetes mellitus. ^a^Number of completed weeks at the end of pregnancy or on admission if undelivered.

Table 2 shows the number of live births, MNM, MD, MNM ratio and SMO ratio for each hospital. Hospital A had the most live births (3515) in the three-month study period, most MNM (150) and the highest MNM ratio (43/1000 live births). The six maternal deaths in hospital A were due to tuberculosis (2), hepatitis E (2), ruptured uterus (1) and septic miscarriage (1). The three maternal deaths in hospital B were due to complicated malaria (2) and a woman who died upon arrival due to an unclear cause. The SMO ratio for all hospitals was 35/1000 live births, 95% CI 30–40.

**Table 2.** For each hospital number of live births, maternal near-miss, maternal deaths, MNM ratio and the SMO ratio.

Facilities	Livebirths	MNM	MD	MNM/ 1000 LB	95% CI	SMO/ 1000 LB	95% CI
Hospital A	3515	150	6	43	36–50	44	37–51
Hospital B	1326	30	3	23	15–31	25	16–33
Hospital C	664	12^a^	0	18	8–28	18	8–28
Hospital D	267	6^a^	0	22	4–40	22	4–40
Total	5772	194	9	34	29–38	35	30–40

^a^Three MNM women from hospital C and one MNM woman from Hospital D were referred to hospital A and part of the total number of these hospitals. MNM, Maternal Near Miss; MD, Maternal Death; LB, livebirths; CI, Confidence Interval; SMO, severe maternal outcome.

### Underlying causes of MNM and MNM criteria

**Figure 1.**
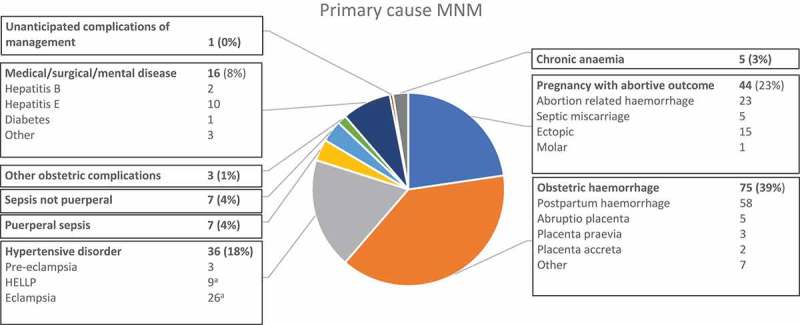
Primary cause of maternal near miss. N = 194. ^a^Two women with eclampsia also had HELLP syndrome and are counted twice.

The underlying cause of the MNM is presented in Figure 1. The commonest causes were obstetric haemorrhage (75, 38.7%, 95% CI 31.8–45.9), abortion-related complications (44, 22.7%, 95% CI 17.0–29.2) and hypertensive disorders (36, 18.6%, 95% CI 13.3–24.8).

Table 3 compares the two sets of MNM criteria and the frequency of each criterion present among the 194 MNM. The frequencies of each criterion separated for the four hospitals are presented in Appendix 2. Of the unique SSA criteria, eclampsia and sepsis were most frequently present with 26 women fulfilling each of these criteria. Eighteen women with eclampsia and 16 women with sepsis did not fulfil any WHO criterion and would have been missed by this set of criteria. Sepsis was caused by endometritis (6), septic miscarriage (3), pyelonephritis (2), wound sepsis (2), abdominal sepsis (2) and pneumonia (1). For these women, sepsis resolved within 24 h after starting antibiotics, wound treatment or evacuation of retained products of conception and therefore were assessed as not severe morbidity. There were two women with uterine rupture of whom one did not fulfil any other WHO criterion. All women with eclampsia and uterine rupture were assessed as severe morbidity. Four women with severe malaria had also either renal, hepatic or neurological dysfunction and this criterion did not contribute to the identification of MNM. The five women who fulfilled the criterion of ‘laparotomy other than caesarean section or ectopic pregnancy’ but no other (WHO) criteria were due to septic cervical ectopic pregnancy not suitable for vaginal approach (Text box 1 case 3), laparotomy after caesarean section to perform B-lynch suture, perforated appendix, septic abdominal pregnancy and transabdominal approach to remove missed miscarriage in second trimester after failed medical induction. All five were assessed as having sustained severe morbidity. Of the unique WHO criteria, ‘bilirubin > 100 mmol/L’ was most frequently present, in 13 women. At the time of study, a hepatitis E outbreak was ongoing and became the underlying disease in ten MNM and two maternal deaths.

**Table 3.** Frequencies of maternal near-miss by type of organ system dysfunction.

MNM identification criteria	Total per MNM criterion N, % (95% CI)	MNM by SSA only	MNM by WHO only
**Severe maternal complications**	**72, 37.3% (30.3–44.3)**		
*Eclampsia*	26	18	
*Sepsis or severe systemic infection*	26	16	
*Uterine rupture*	2	1	
*Pulmonary oedema*	7	2	
*Severe abortion complications*	8	3	
*Severe malaria*	4	0	
*Severe pre-eclampsia with ICU admission*	4	1	
*Laparotomy other than caesarean section or ectopic pregnancy*	12	5	
**Cardiovascular dysfunction**	**21, 10.8% (6.8–16.1)**		
Shock	20		
Cardiac arrest	0		
Use of continuous vasoactive drugs	3		0
CPR	0		
pH < 7.1	1		1
Lactate >5 mEq/mL	0		0
**Respiratory function**	**16, 8.2% (4.8–13.0)**		
Acute cyanosis	0		
Gasping	0		
Respiratory rate >40 or <6/min	5		
Intubation and ventilation for ≥60 minutes not related to anaesthesia	11		
Oxygen saturation <90% for ≥60 minutes	4		
**Renal dysfunction**	**4, 2.1% (0.6–5.2)**		
Oliguria non-responsive to fluids or diuretics	4		
Creatinine ≥300 µmol/l or ≥3.5 dL/dL	1		
Dialysis for acute renal failure	1		0
**Coagulation/hematologic dysfunction**	**147, 75.8% (69.1–81.6)**		
Failure to form clots	6		
Transfusion of *≥ 2 units* of red blood cells	140	103	
Acute thrombocytopenia (<50,000 platelets/ml)	12		
**Hepatic dysfunction**	**13, 6.7% (3.6–11.2)**		
Jaundice in the presence of pre-eclampsia	0		
Bilirubin >100 mmol/l or >6.0dL/dL	13		9
**Neurological dysfunction**	**8, 4.1% (1.8–8.0)**		
Loss of consciousness lasting 12 hours (GCS < 10)	6		
Stroke	0		
Loss of consciousness and ketoacids in urine	0		
Uncontrollable fit	1		
New onset of paralysis	1		
**Uterine dysfunction**	**6, 3.1% (1.1–6.6)**		
Hysterectomy following infection or haemorrhage	6		
Total	194	133	10

*In italics* unique SSA criteria and underlined unique WHO criteria. A woman could fulfil more than one MNM criterion. At the top of each section the total number of women with each organ dysfunction, followed by the proportion, calculated by the total divided by 194 MNM women and presented as percentage. The second and third column presents the number of women that only fulfilled criteria of either the WHO or SSA set and would have been missed if the other set was applied. MNM, Maternal near miss; CI, Confidence interval; SSA, sub-Saharan Africa; WHO, World Health organization, ICU, intensive care unit; CPR, cardiopulmonary resuscitation; GCS, Glasgow coma scale.

**Figure 2.**
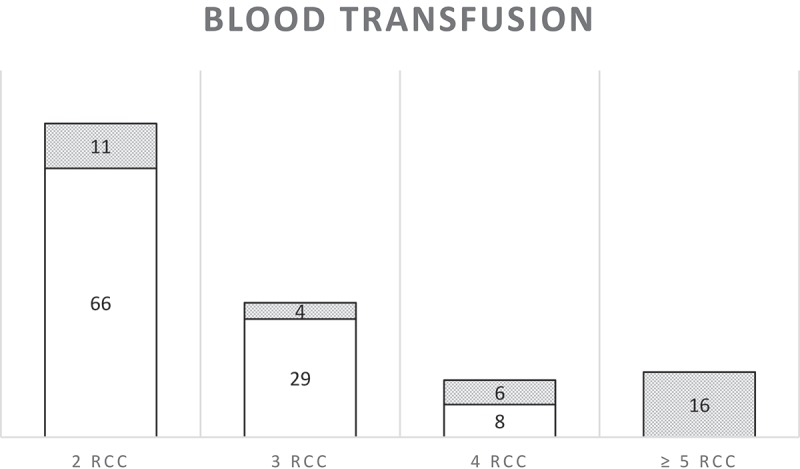
Summary of number of women who received two or more units of blood transfusion. In white the women that did not fulfil another criterion of the WHO set and would have been missed by the WHO MNM criteria. In grey the number of women that did fulfil another criteria of the WHO MNM criteria. RCC, red cell concentrate.

Figure 2 summarizes the number of units of blood given to MNM. SSA MNM criterion of at least two units of blood was fulfilled by 140 women. An estimation of the amount of blood loss was not available in medical records. Assessment of severity of haemorrhage was based on the vitals of the patients and interventions performed to stop the bleeding. Two or three units of red cells were given to 95 women for mild/moderate haemorrhage or chronic anaemia who did not fulfil any WHO criterion and were not considered to be MNM. Fourteen women received four units and were assessed to be MNM from severe haemorrhage. Even though these women did not reach the clinical thresholds of shock, they often had tachycardia and needed several interventions to stop the bleeding and to restore these clinical parameters (Text box 1 case 3 and 4). Of the women identified through the register of blood bank, 20 women received two units of blood and eight women three.

If only the WHO criteria would have been applied to our cohort 61 MNM would have been identified and if the SSA criteria were applied 184 MNM. In Textbox 1, several summaries are presented of MNM that would have been missed if the WHO MNM criteria were applied.

### Long-term outcome

Although 194 women survived the pregnancy-related complication, 15 (7.7%) women sustained a long-term complication. Six (3.1%) women underwent hysterectomy of whom five were haemorrhage and one infection related. Three (1.5%) women developed cardiomyopathy and three needed a colostomy: one woman had iatrogenic bowel perforation during caesarean section, one had an abdominal pregnancy with placental invasion into the sigmoid and one woman with a previous caesarean section had an obstructed bowel due to adhesions during her current pregnancy. Two (1.0%) women had a ruptured uterus due to obstructed labour of whom one developed a vesicovaginal fistula. One women with known chronic renal failure developed acute on chronic renal failure secondary to pre-eclampsia and was still in need of dialysis after discharge. The two women with a uterine rupture, two women who needed a colostomy and one woman with a haemorrhage-related hysterectomy had had one or more previous caesarean sections.

## Discussion

In the middle-income setting of Namibia, both the WHO and SSA MNM criteria cannot be applied without amendments. Even though the WHO developed the criteria to be applied across settings, our findings suggest it would result in an underestimation of MNM in Namibia, which corresponds with experiences from low-income settings [11,12,16]. The SSA criteria were proposed for use in low-income settings. Application of the SSA criteria to our cohort resulted in an overestimation of MNM which suggests that both sets are less feasible to be applied to a middle-income setting such as Namibia. There are several other middle-income countries which used the MNM approach. However, they applied the WHO criteria or locally established criteria without validating them, and most of these studies only included referral or tertiary hospitals [16–22]. For example, a large national study from Brazil included 27 referral hospitals and used the WHO criteria [18]. India developed its own MNM criteria and performed a pilot in six tertiary facilities [22]. A recently published systematic review assessed the applicability of WHO criteria in sub-Saharan Africa and included 15 reports of which four reported findings from middle-income countries [16,17,23–25]. These four reports were all from tertiary facilities with availability of resources to apply the WHO criteria [17,23–25].

For MNM registration in all Namibian hospitals of all levels, we decided to adopt the WHO criteria with the addition of the criteria eclampsia, uterine rupture, laparotomy other than caesarean section or ectopic pregnancy’ and a lower threshold of four units of blood. Even though eclampsia and uterine rupture are not part of the WHO criteria, high case fatality rates are reported for both conditions across settings [26–28]. Furthermore, these are commonly used in both high- and low-income countries to define MNM for local and national registrations and recently suggested as a standard outcome to define severe morbidity in maternal health-related research [11,29–32]. To our knowledge, the criterion ‘Laparotomy other than caesarean section or ectopic pregnancy’ is not commonly used to define MNM. However, in our cohort, several women would have been missed and this criterion, therefore, turned out to be a useful addition. We opted to change the threshold for blood transfusion to four units of red cells. The threshold of two or three units resulted in inclusion of many women with minor to moderate haemorrhage-related complications, as well as inclusion of women with chronic anaemia. At a threshold of five units of blood, women with severe haemorrhage would have been missed.

The criterion sepsis led to overinclusion in our cohort of women who were not critically ill. The SSA set used a definition of sepsis developed for the non-obstetric population [33]. Amendment of the definition of sepsis, with higher thresholds of clinical signs, might lead to better MNM identification. The remaining SSA criteria (pulmonary oedema, severe abortion complications, severe malaria and severe pre-eclampsia with ICU admission), appeared not to be essential to identify MNM, resulted in inclusion of women with minor morbidity or were unclear leading to incorrect inclusion.

The prevalence of SMO is expected to correlate with the MMR of a country [6]. The WHO multicountry survey showed an SMO ratio of 15.9/1000 livebirths in countries with a comparable MMR [15]. By applying the SSA criteria we found a more than double SMO ratio, whereas by applying the above-suggested amendments we would have identified 93 MNM, which leads to an SMO ratio of 17.5/1000 live births.

We recommend other middle-income countries to validate our criteria in their setting. A country’s income level does not necessarily correlate with maternal outcome [34]. For example, Namibia has a relatively high prevalence of severe maternal outcome. The income level is based on national income per person and does not take into account income inequities [14]. However, a country’s income level will, in most cases, affect the availability of resources. This poses the most important restriction in use of the WHO MNM criteria.

Access to care does not seem to be a major issue contributing to poor maternal outcome in Namibia. The country has one of the best road networks in Africa and an effective referral system appears to be in place. This is reflected by the high antenatal care attendance rate of 96% and the fact that 87% of women give birth in health facilities, attended by a skilled health-care worker [9]. A recent national maternal death review, of which the report is expected to be published next year, indicates that most deaths occur whilst the woman is in the health facility. Namibian health facilities, like most state-funded facilities in sub-Saharan Africa, have high turnover of staff, and are staffed mainly by junior staff with limited experience and supervision. Within the five-step ‘obstetric transition’ as described by the World Health Organization, Namibia is now in stage III, the typical state of a middle-income country that has largely overcome barriers for women to access care, where women are now indeed reaching health facilities, and hence where improved quality of care becomes the critical step to achieve a reduction of maternal mortality [35].

Over 80% of the underlying causes of the MNM were direct pregnancy-related causes. We feel that this might be an overrepresentation, caused by the over-reporting of haemorrhage. A better representation of the underlying causes is expected to be found in the planned national MNM observational study with the amended criteria. Abortion-related complications were the second most common underlying cause of the MNM. In Namibia, women only have limited access to abortion and only for significant maternal or fetal indications. A high incidence of abortion-related MNM events is seen in other countries where women have limited access to safe abortion as well [36].

A considerable proportion of our women had a previous caesarean section and over half of our women gave birth by caesarean section which correlates with other reports [15]. A possible explanation for this high proportion could be our sick population. However, even in an MNM population, there are reports available indicating a high number of caesarean sections without medical indication and for some women, the MNM event was the direct result rather than the indication for caesarean section [37]. In our cohort, a woman sustained iatrogenic bowel injury during birth by caesarean section and needed several laparotomies and went home with a colostomy.

Our study has several limitations. We used a relatively small cohort and were unable to perform a sample size calculation. Possibly larger studies, such as the planned national MNM observational study, may permit additional conclusions with statistical significance. Availability of background data was very limited regarding maternal morbidity and mortality in Namibia. Objective data such as case fatality rate would have been useful to further support our decisions to amend the WHO criteria.

Data were collected by local staff who already had a high workload and MNM might have been missed. Even though data collection done by full-time research staff might have been more accurate, we aimed for data collection mainly done by local staff as this would enable us to identify barriers and challenges for a planned routine national MNM data collection in all Namibian hospitals. The project was overall well received by staff members as they felt they could finally report ‘the other side of the story, rather than only reporting maternal deaths’. The research team identified an additional 31 women, of whom the majority appeared to not be critically ill. This further supports the experience that our staff were motivated to report MNM. We found a higher prevalence of MNM in the capital. As the national referral centre, this was expected. However, this is most likely also caused by reporting bias since this was the only facility where data collection was supported by the research team. We suggest the assessment of long-term complications should become part of all MNM projects. Even though these women nearly died but survived a pregnancy-related complication, a considerable proportion left the hospital with significant disability.

## Conclusions

This report validated MNM criteria to be applied nationwide in hospitals of all levels in a sub-Saharan African country. Our findings suggest that a middle-income country such as Namibia needs MNM criteria ‘in between’ the WHO and SSA criteria. Context-specific MNM criteria are vital to help Namibia and other middle-income countries to identify MNM. Only with accurate MNM criteria can this tool be used for what it is designed for; to improve maternity care and to stop women dying of avoidable causes. We recommend other middle-income countries to validate our criteria in their setting when the WHO criteria are not feasible, rather than developing new criteria, as this will allow comparison of findings.

**Text box 1.** Summary of cases missed by WHO criteria sorted by assessment of research team as severe or not severe morbidity case. CS, caesarean section; G, gravida; P, parity; Hb, haemoglobin; WBC, white blood cells x 10^9^/L; RCC, red cell concentrate.

**Severe morbidity**
Eclampsia
(1) A 17-year old primigravida fitted eight times due to eclampsia at home and in a small district hospital 119 km away from hospital B. She was referred to hospital B were a CS was done. She recovered well.
Uterine rupture
(2) A 28-year old G3P2 with one previous CS was admitted in active labour in hospital A. An emergency CS was done and a uterine rupture found. A fresh stillborn baby was delivered. The woman developed a vesicovaginal fistula and received 1 unit of blood during admission.
Laparotomy other than CS or ectopic
(3) A 33-year old G3P2 presented to Hospital A with a septic cervical pregnancy. A hysterotomy, abdominal approach, was done to remove the pregnancy since a vaginal approach was deemed not to be feasible. To control the bleeding a bilateral ligation of the uterine arteries was done. The woman did not fulfil the criteria of shock. She received four units of blood and two units fresh frozen plasma.
Transfusion of 2 or more units of blood
(4) A 21-year old primigravida presented to hospital D with severe bleeding due to an incomplete miscarriage. Her Hb was 5.2 g/dL, she received four units of blood and the retained products of conception were removed.
**No severe morbidity**
Sepsis
(1) A 27-year old woman developed fever 1 day after her normal delivery in hospital A. She had a heart rate of 100/min, WBC of 9. She was not acutely ill, endometritis was suspected and she recovered well on intravenous antibiotics.
Transfusion of 2 or more units of blood
(2) A 28-year old G5P4 presented to hospital A with symptomatic anaemia at 32 weeks of gestation. Her Hb was 6.8 g/dL and she was transfused 2 RCCs.
(3 A 17-year old primigravida gave birth in Hospital B with minimal blood loss. During antenatal care an Hb of 5.9 g/dl was found. Three units of blood were transfused postpartum.

## Data Availability

Data supporting the findings are available upon reasonable request.

## Paper context

The Maternal Near Miss (MNM) approach was introduced to improve maternal health. A recognized set of MNM identification criteria will facilitate comparisons across settings. ‘WHO MNM criteria’, designed to be applied across all settings, underestimate and ‘sub-Saharan Africa MNM criteria’, an adaptation for use in low-income settings, overestimate the prevalence of MNM in a middle-income country such as Namibia, where MNM criteria ‘in between’ may be more appropriate. Adaptations for middle-income countries were proposed.

## Appendices

**Appendix 1.** Comparison of WHO MNM criteria and SSA MNM criteria.

WHO near-miss criteria	Sub-Saharan Africa near-miss criteria
**Clinical criteria**	**Clinical criteria**
Acute cyanosis	Acute cyanosis
Gasping	Gasping
Respiratory rate >40 or <6/min	Respiratory rate >40 or <6/min
Shock^a^	Shock
Oliguria non-responsive to fluids or diuretics^b^	Oliguria non-responsive to fluids or diuretics
Failure to form clots	Failure to form clots
Loss of consciousness lasting >12 h	Loss of consciousness lasting 12 hours
Cardiac arrest	Cardiac arrest
Stroke	Stroke
Uncontrollable fit / total paralysis	Uncontrollable fit / total paralysis
Jaundice in the presence of pre-eclampsia	Jaundice in the presence of pre-eclampsia
**Laboratory-based criteria**	**Laboratory-based criteria**
Oxygen saturation <90% for ≥60 minutes	Oxygen saturation <90% for ≥60 minutes
PaO2/FiO2 <200 mmHg	
Creatinine ≥300µmol/l or ≥3.5mg/dL	Creatinine ≥300µmol/l or ≥3.5mg/dL
Bilirubin >100mmol/l or >6.0 mg/dL	
pH < 7.1	
Lactate >5 mEq/mL	
Acute thrombocytopenia (<50,000 platelets/ml)	Acute thrombocytopenia (<50,000 platelets/ml)
Loss of consciousness and ketoacids in urine	Loss of consciousness and ketoacids in urine
**Management-based criteria**	**Management-based criteria**
Use of continuous vasoactive drugs	
Hysterectomy following infection or haemorrhage	Hysterectomy following infection or haemorrhage
Transfusion of ≥5 units of red cells	Transfusion of ≥ 2 units of red cells
Intubation and ventilation for ≥60 minutes not related to anaesthesia	Intubation and ventilation for ≥60 minutes not related to anaesthesia
Dialysis for acute renal failure	
Cardio-pulmonary resuscitation	Cardio-pulmonary resuscitation
	Laparotomy other than caesarean section or ectopic pregnancy
**Severe maternal complications**	**Severe maternal complications**
	Eclampsia
	Sepsis or severe systemic infection^c^
	Uterine rupture^d^
	Pulmonary oedema
	Severe abortion complications^e^
	Severe malaria^f^
	Severe pre-eclampsia with ICU admission

MNM, Maternal near-miss; SSA, sub-Saharan Africa; WHO, World Health organisation, ICU, intensive care unit; CPR, cardio pulmonary resuscitation; GCS, Glasgow coma scale. ^a^persistent systolic blood pressure <80 mmHg or a persistent systolic blood pressure <90 mmHg with a pulse rate at least 120 bpm, ^b^urinary output <30 ml/hour over 4 hours or <400 ml/24 hr, ^c^presence of fever (Temp >38°C) AND confirmed or suspected infection AND at least one of the following: heart rate >90, respiratory rate >20, WBC > 12 or < 4 x 10^9^/L, ^d^complete rupture of uterus during labour confirmed by laparotomy, ^e^septic incomplete abortion, complicated gestational trophoblastic disease with anaemia, ^f^major signs of organ dysfunction and/or high level parasitaemia or cerebral malaria.

**Appendix 2.** Frequencies of maternal near-miss by type of organ system dysfunction in the four hospitals

	Facilities				
	A	B	C	D	Total
**Severe maternal complications**	**52**	**16**	**4**	**0**	**72, 37.3%**
*Eclampsia*	17	7	2	0	26
*Sepsis or severe systemic infection^b^*	22	2	2	0	26
*Uterine rupture^c^*	1	1	0	0	2
*Pulmonary oedema*	5	2	0	0	7
*Severe abortion complications^d^*	7	1	0	0	8
*Severe malaria^e^*	0	4	0	0	4
*Severe pre-eclampsia with ICU admission*	1	3	0	0	4
*Laparotomy other than caesarean section or ectopic pregnancy*	12	0	0	0	12
**Cardiovascular dysfunction**	**17**	**2**	**2**	**0**	**21, 10.8%**
Shock^f^	16	2	2	0	20
Cardiac arrest	0	0	0	0	0
Use of continuous vasoactive drugs	3	0	0	0	3
CPR	0	0	0	0	0
pH < 7.1	1	0	0	0	1
Lactate >5 mEq/mL	0	0	0	0	0
**Respiratory function**	**14**	**2**	**0**	**0**	**16, 8.3%**
Acute cyanosis	0	0	0	0	0
Gasping	0	0	0	0	0
Respiratory rate >40 or <6/min	4	1	0	0	5
Intubation and ventilation for ≥60 minutes not related to anaesthesia	10	1	0	0	11
Oxygen saturation <90% for ≥60 minutes	4	0	0	0	4
**Renal dysfunction**	**3**	**1**	**0**	**0**	**4, 2.0%**
Oliguria non-responsive to fluids or diuretics^g^	3	1	0	0	4
Creatinine ≥300µmol/l or ≥3.5mg/dL	1	0	0	0	1
Dialysis for acute renal failure	1	0	0	0	1
**Coagulation/hematologic dysfunction**	**112**	**24**	**6**	**5**	**147, 75.8%**
Failure to form clots	4	2	0	0	6
Transfusion of *≥ 2 units* of red blood cells	108	21	6	5	140
Acute thrombocytopenia (<50,000 platelets/ml)	8	4	0	0	12
**Hepatic dysfunction**	12	1	0	0	**13, 6.7%**
Jaundice in the presence of pre-eclampsia	0	0	0	0	0
Bilirubin >100mmol/l or >6.0 mg/dL	12	1	0	0	13
**Neurological dysfunction**	**6**	**2**	**0**	**0**	**8, 4.2%**
Loss of consciousness lasting 12 hours (GCS < 10)	4	2	0	0	6
Stroke	0	0	0	0	0
Loss of consciousness and ketoacids in urine	0	0	0	0	0
Uncontrollable fit	1	0	0	0	1
New onset of paralysis	1	0	0	0	1
**Uterine dysfunction**	**5**	**1**	**0**	**0**	**6, 3.0%**
Hysterectomy following infection or haemorrhage	5	1	0	0	6
**Total**	**150**	**30**	**9**	**5**	**194**

Italics unique SSA criteria and underlined unique WHO criteria. A woman could fulfil more than one MNM criterion. At the top of each section the total number of women with each organ dysfunction, followed by the proportion, calculated by this total divided by 194 MNM women and presented as percentage. MNM, Maternal near miss; SSA, sub-Saharan Africa; WHO, World Health organisation, ICU, intensive care unit; CPR, cardio pulmonary resuscitation; GCS, Glasgow coma scale. ^b^presence of fever (Temp >38°C) AND confirmed or suspected infection AND at least one of the following: heart rate >90, respiratory rate >20, WBC > 12 or < 4 x 10^9^/L; ^c^complete rupture of uterus during labour confirmed by laparotomy; ^d^septic incomplete abortion, complicated gestational trophoblastic disease with anaemia; ^e^major signs of organ dysfunction and/or high level parasitaemia or cerebral malaria; ^f^persistent systolic blood pressure <80 mmHg or a persistent systolic blood pressure <90 mmHg with a pulse rate at least 120 bpm; ^g^urinary output <30 ml/hour over 4 hours or <400 ml/24 hr.
